# Optimising the photothrombotic model of stroke in the C57BI/6 and FVB/N strains of mouse

**DOI:** 10.1038/s41598-022-11793-6

**Published:** 2022-05-09

**Authors:** Adriana Knezic, Brad R. S. Broughton, Robert E. Widdop, Claudia A. McCarthy

**Affiliations:** Department of Pharmacology, Cardiovascular Disease Program, Biomedicine Discovery Institute, Monash University, Clayton, VIC Australia

**Keywords:** Stroke, Experimental models of disease

## Abstract

The photothrombotic stroke model relies on the interaction between photosensitive-dye and light for clot formation. Interestingly, the relationship between the length of light exposure and stroke-outcome has never been examined. This model has yet to be established in the FVB/N strain, even though stroke-outcomes are strain-specific. Therefore, this study aimed to examine the effect of different lengths of light exposure in two strains of mice on photothrombotic stroke. Male FVB/N and C57Bl/6 mice were subjected to stroke using 15, 18, or 20-min light exposure. Mice underwent functional testing for up to 7 days. Infarct volume was assessed with thionin staining, and cellular responses to injury analysed via immunofluorescence at 7-days post-stroke. Blood brain barrier (BBB) breakdown was assessed using Evans blue dye at 4.5-h post-stroke. Increasing light exposure from 15 to 20-min increased infarct volume but not functional deficit. Interestingly, there were strain-specific differences in functional outcomes, with FVB/N mice having less deficit on the hanging wire test than C57BI/6 after 15-min of light exposure. The opposite was seen in the adhesive removal test. There was no difference in the number of neurons, astrocytes, microglia, macrophages, and T cells between the strains, despite FVB/N mice demonstrating greater BBB breakdown and an enlarged spleen post-stroke. Increasing light exposure systematically increases infarct volume but does not worsen functional outcomes. FVB/N and C57Bl/6 mice exhibit subtle differences in functional outcomes post stroke, which highlights the need to choose tests which are appropriate for the mouse strain being used.

## Introduction

Stroke is a leading cause of both disability and death world-wide^1^. Despite having such a large impact on patients and an economic burden, there are very limited treatment options for patients. Therefore, there is a large need for the development of new therapies to treat this debilitating condition. Animal stroke models have allowed research and development of new therapies, however, none of these models are a perfect representation of human stroke and there has been a failure to translate these animal studies clinically^2^. Given that stroke is such a heterogenous disease, using a wider range of animal models and species/strains of animals has been recommended to improve these translatability issues^3^. The filament model of middle cerebral artery occlusion (MCA) is the most commonly used stroke model and is advantageous in that it closely mimics the location of human stroke^4^. However, this stroke model is very severe and has large variation in infarct size. The photothrombotic (PT) model of stroke induces focal cerebral ischemia. This method involves injecting animals with the photo-sensitive dye, rose bengal, and then illuminating the skull to cause free radical formation, resulting in damage to the endothelium, platelet aggregation and blood clot formation^5^. An advantage of this model is that it has higher reproducibility compared to the filament model of MCAO. However, PT stroke produces a small or no ischemic penumbra^6^ which is a limitation as human stroke generally features a large penumbral area. Furthermore, the lack of a penumbral region may render PT stroke more resistant to neuroprotective strategies^7,8^. While the PT model is increasingly being used, there is no standardised protocol, such that the length of light exposure varies depending on the protocol being followed and typically ranges between 15 and 20 min^5,9–11^. Surprisingly, the relationship between the length of light exposure and stroke-induced damage has not yet been examined in detail.

Additionally, it has been shown in the filament model of MCAO there are differences in functional outcomes and the immune response between C57Bl/6 and FVB/N mice, with C57Bl/6 mice having a greater T helper(Th)-1 compared to Th-2 response, whereas the opposite is seen in FVB/N mice^12^. Furthermore, FVB/N mice have a greater macrophage and neutrophil response compared to C57Bl/6 mice. This translates into FVB/N mice having less functional impairment than C57Bl/6 mice following MCAO. However, there are no reports on the effects of photothrombosis in the FVB/N strain of mice, and therefore it remains to be established whether the strain differences identified in the filament MCAO model of stroke are also evident in this milder model of cerebral ischemia.

Patients experience a breakdown of the blood–brain barrier (BBB) following stroke^13^. Given that the BBB protects the brain against toxins and maintains brain hemodynamics, its disruption results in increased edema and the potential for hemorrhagic transformation^14^. However, this breakdown can also be beneficial given that it allows drugs access to the brain which are not normally permeable across the BBB. In rats following PT stroke, BBB breakdown has been shown to occur as early as 1 h post-stroke^15^, and increases at 3-days post-stroke^16^. In mice, disruption of the BBB has also been shown to occur around the peri-infarct area as soon as 3 h^17^, and are still seen at 3 days post-stroke^18^, BBB disruption was maintained up to 21 days post-stroke in the PT model^19^. Interestingly, there is very limited characterisation of BBB breakdown in the PT model of stroke, and potential strain differences in the BBB response to stroke have never been examined in any model of cerebral ischemia.

Therefore, the aims of the current study were to establish the relationship between the length of light exposure and damage in the PT model, and to characterise the differences in functional outcomes, infarct size, cellular response and BBB breakdown between C57Bl/6 and FVB/N mice in this model of stroke.

## Materials and methods

### Animals

All experimental procedures for this study were approved by a Monash University Ethics Committee (MARP/2017/144) and (MARP/23298) and performed in accordance with the National Health and Medical Research Council of Australia and ARRIVE guidelines for the care and use of animals in research. A total of 65 male FVB/N and 47 male C57Bl/6 mice (8 to 12 weeks old) obtained from the Monash Animal Research Laboratory were included in this study (Monash University, Clayton campus). All mice were maintained on a 12-h light/dark cycle with free access to water and food pellets and were housed in specific pathogen free cages.

### Inclusion criteria and blinding

To be included in the current study, mice needed to survive for the 7 days post stroke and to have a measurable infarct. There are anatomical differences in the shape of the head between the FVB/N and C57Bl/6 strains of mice, therefore positioning in the stereotaxic frame and light source placement required optimisation for the FVB/N strain in order to produce a successful stroke. The mice used during this optimisation protocol account for a large number of mice that were excluded. In total, 26 mice were excluded as they did not meet the criteria or were euthanised at an earlier time point due to animal welfare.

Experimenters were blinded to all groups.

### Photothrombotic stroke surgery

The PT stroke surgery was performed as previously described by Labat-gest and Tomasi^6^. However, to obtain a concentration–response to light exposure, the light source was turned on for either 15, 18, or 20 min in both the C57Bl/6 and FVB/N strains of mice. Sham mice were subjected to the same procedure but did not receive the intraperitoneal (i.p) injection of rose Bengal. For a full description of the PT surgical procedure, refer to the Supplementary Information.

### Functional tests

The hanging wire and adhesive removal tests were performed as an indicator of functional outcome. These tests were performed the day prior to stroke induction, to establish baseline performance, and then repeated at 24 h, 72 h, & 7 days post-stroke.

### Hanging wire test

The hanging wire test was used to assess forelimb strength and grasping ability, providing an indication of motor function. The test was performed as described by Hattori, et al.^20^, where mice were suspended by their forelimbs on a wire between two posts 45 cm above a foam pillow. The time in seconds (s) for the mice to fall from the wire was recorded with a maximum test period of 300 s. The test was repeated three times per mouse, with a 5-min break period between each test. An average of the 3 individual tests was calculated and recorded as the latency to fall for that time point.

### Adhesive removal test

The adhesive removal test was used to measure sensory function and motor coordination^21^. Mice were placed in an empty cage and allowed to acclimate for 2 min. Following this time, an adhesive sticker (8 mm diameter) was placed on each forelimb paw. The mice were then placed back in the empty cage and the time for the mouse to remove both adhesives was recorded. The maximum test period was 300 s. The test was repeated three times per mouse with a 5-min rest period between tests. The average of the 3 individual test periods was calculated and recorded as the time to remove adhesive for that testing period.

### Harvesting of tissues

On day 7 post-stroke, mice were euthanised with inhaled anaesthetic isoflurane (in 2–4% oxygen; Issorane, Baxter, Baxter Healthcare Pty. Ltd). Brains were removed from the skull and snap frozen using liquid nitrogen. The spleen and thymus were then removed, weighed, and snap frozen using liquid nitrogen. Tissue samples were then stored at − 80 °C. Spleen and thymus weight ratio was calculated using the formula: organ weight(mg)/body weight(g).

### Preparing tissue sections

Brain sections were cut using a cryostat (Leica Biosystems) and were thaw-mounted onto Superfrost Plus glass slides (Thermo Scientific; 25 × 75 × 1 mm) and stored at − 80 °C. To assess infarct volume, 30 µm thick coronal sections were collected every 240 µm across the span of the infarct. For immunofluorescence, a series of twelve 10 µm thick coronal sections were collected every 240 µm within the infarct.

### Infarct size analysis

Thionin staining was used to measure infarct volume. Thionin stains Nissl substances in soma and dendrites, where neuronal bodies stain dark blue, neuropil stains purple, whilst the infarcted tissue does not retain stain and appears white^22^. Brain tissue sections (30 µm thick) were stained with 0.1% thionin (Sigma, Australia, diluted in acetic acid, Ajax Chemicals, Australia), for 2 min, rinsed in dH_2_O water for 5 s, placed in 70% and 100% ethanol for 2 min each, and then coverslipped with xylene (Merck, Australia) and distyrene plasticiser (DPX; Merck, Australia).

The sections were then photographed using a charge coupled device (CCD) camera (Cohu Inc., San Diego, CA, USA) mounted above a lightbox (Biotec-Fischer Colour Control 5000, Reishkirchin, Germany) using IS capture imaging software. Infarct volume was analysed using image analysis software (Image J, version 1.52a, NIH, Bethesda, MD, USA, available at https://imagej.nih.gov/ij/download.html). The area of infarct for each section was measured and the sum of the total infarct area for all the stained sections was multiplied by the distance between the sections to give an approximation of the total infarct volume. Edema was corrected for using the following formula: Corrected infarct area = [left hemisphere area – (right hemisphere area – right hemisphere infarct area)]^23^.

### Immunofluorescence

For immunohistochemical analysis, 10 µm thick tissue sections were taken from mice subjected to either sham surgery or 18 min of photothrombosis. The 18-min light exposure was selected as it produced a sufficient functional deficit in both strains, with the area of damage still being conserved to the cortex. Cellular architecture was assessed using a: mouse anti-NeuN antibody (MAB377, 1:500 dilution; Merck Millipore, USA) to detect the number of viable neurons; goat anti-GFAP antibody (SAB2500462, 1:500 dilution; Sigma) to detect astrocytes; rat anti-F4/80 antibody (MCA497G, 1:100 dilution, Bio-Rad) to detect macrophages; rabbit anti-CD3 antibody (Ab16669, 1:200 dilution, Abcam) to detect T cells; goat anti-Iba1 antibody (Ab5076, 1:400 dilution, Abcam) to detect microglia in the brain following stroke.

NeuN, GFAP, and Iba1 images were captured at 400X magnification using an Olympus fluorescent microscope and CellSens software (Version 1.15; Olympus, USA), 3 images were taken from the infarct region, peri-infarct region, and undamaged region of the stroked hemisphere, and also from the non-stroke hemisphere, in the region corresponding to the infarcted area. For mice subjected to sham-surgery, the region that would be subjected to infarct was duplicated as the peri-infarct region as they are in the same brain region. The number of NeuN positive ( +) cells per region were counted using Image J (version 1.52a, NIH, Bethesda, MD, USA, available at https://imagej.nih.gov/ij/download.html) and averaged per region of the brain for each animal. The percentage area of positive staining of GFAP or Iba1 was measured using Image J (version 1.52a, NIH, Bethesda, MD, USA, available at https://imagej.nih.gov/ij/download.html) and averaged per region of the brain for each animal.

For CD3 staining, each hemisphere of the brain was imaged and the total number of CD3 positive cells was counted per hemisphere due to a low number of cells in the brain.

For F4/80, the stroked and non-stroked hemisphere was imaged at 40X magnification, and the percentage area of positive staining was measured with Image J (version 1.52a, NIH, Bethesda, MD, USA, available at https://imagej.nih.gov/ij/download.html) and averaged per region for each animal.

For detailed immunofluorescence protocols and an image of the different brain regions analysed, refer to Supplementary Fig. S1 online. Representative images can be found in the online Supplementary material, Fig. S3 to S5.

### Evans blue

To assess permeability of the BBB, mice were subjected to 18 min of PT stroke or sham surgery and were then injected with 2% Evans Blue dye (Sigma) in saline (4 mL/KG) at 4 h post-stroke (i.v). The dye was allowed to circulate for 30 min prior to culling. The animals were then euthanised and transcardially perfused with heparinised PBS (10u/mL; Heparin sodium, Hospira, Australia). The brain was removed and imaged.

To quantify the amount of evans blue staining, the % area of blue dye in the hemisphere was measured by tracing the area around the ipsilateral hemisphere and the area of evans blue extravasation in Image J (version 1.52a, NIH, Bethesda, MD, USA, available at https://imagej.nih.gov/ij/download.html). The % area was calculated by: area evans blue extravasation/total area of the ipsilateral hemisphere X 100.

### Statistical analysis

All data was expressed as mean ± standard error of the mean (SEM). All statistical analyses were conducted using GraphPad Prism (version 9, GraphPad Software Inc., USA). Functional tests and weight measurements were analysed using two-way repeated measures (RM) ANOVA, followed by Tukey’s and Sidak’s post hoc tests. To assess evans blue extravasation, a one-way ANOVA test was used with Tukey’s post hoc test. To assess infarct volume and immunofluorescence, a two-way ANOVA was used, with Tukey’s and Sidak’s post hoc tests. Probability values of P < 0.05 were considered as statistically significant.

## Results

### Increasing the length of light exposure increases infarct volume in both strains of mouse

**Figure 1:**
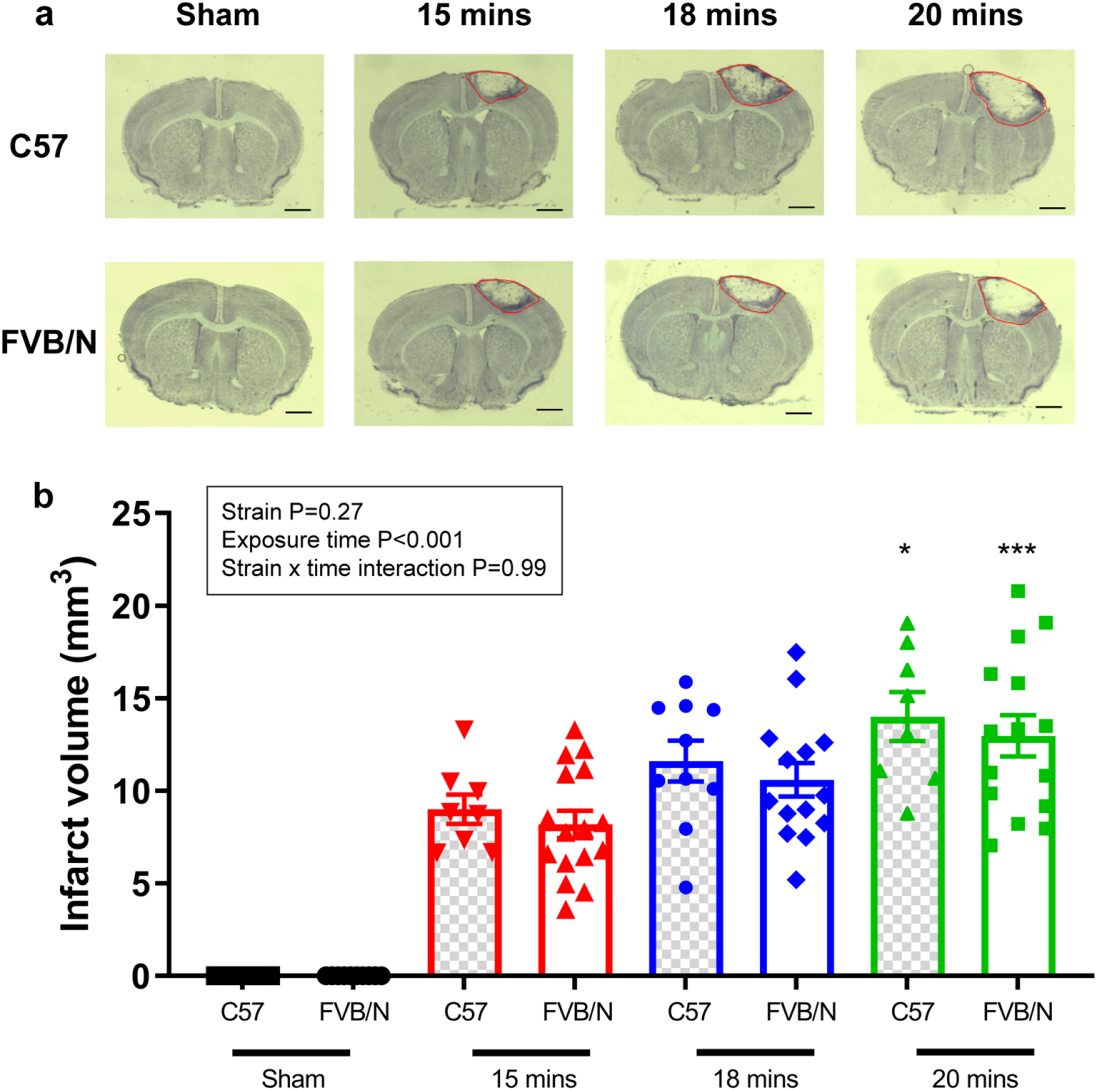
(**a**) Representative images of thionin stained coronal brain sections and infarct volumes mm^3^ (**b**) from either sham operated non-stroked animals (C57Bl/6 n = 11, FVB/N n = 10), or animals with light exposures of 15 min (C57Bl/6 n = 8, FVB/N n = 16), 18 min (C57Bl/6 n = 10, FVB/N n = 14), and 20 min (C57Bl/6 n = 8, FVB/N n = 15) during stroke induction. Infarct area signified with a red line. Data are presented as mean ± SEM; *P < 0.05, ***P < 0.001 vs 15 min in respective group, two-way ANOVA followed by Tukey’s multiple comparisons test. Scale bar represents 1 mm.

There was a relationship between the length of light exposure and infarct volume (Fig. 1, P < 0.001). Indeed, mice given 20-min of light exposure had a significantly larger infarct volume compared to those with a 15-min light exposure (C57Bl/6 15-min: 9.003mm^3^ ± 0.79mm^3^ vs. 20-min: 14.02mm^3^ ± 1.31mm^3^; FVB/N 15-min: 8.19mm^3^ ± 0.74mm^3^ vs. 20-min: 12.97mm^3^ ± 1.11mm^3^; Fig. 1b, P < 0.001). Interestingly, there was no difference in the infarct volume between the FVB/N and C57BI/6 strains of mice.

### C57Bl/6 mice have greater motor impairment and stroke-induced weight loss than FVB/N mice. In contrast, FVB/N mice are more sensitive to the adhesive removal test

**Figure 2:**
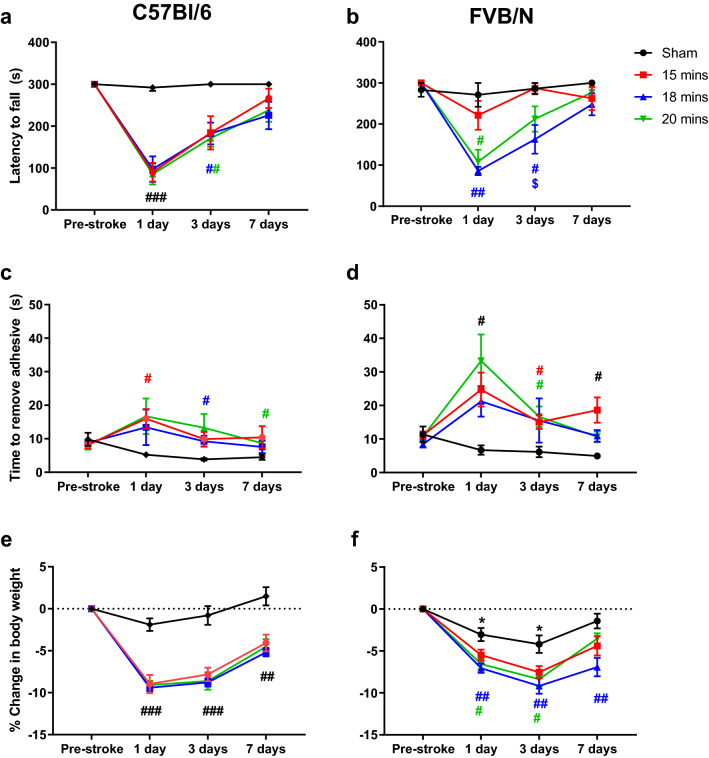
C57Bl/6 mice have greater motor impairment and stroke-induced weight loss compared to FVB/N mice. In contrast, FVB/N mice are more sensitive to the adhesive removal test. Effects of light exposures of 15 (C57Bl/6 n = 8, FVB/N n = 16), 18 (C57Bl/6 n = 10, FVB/N n = 14), and 20 min (C57Bl/6 n = 8, FVB/N n = 15) during stroke induction, prior to stroke, and 1, 3, and 7 days after stroke on: The hanging wire test in C57Bl/6 mice (**a**) or FVB/N mice (**b**), expressed as latency to fall (s); the adhesive removal test for C57Bl/6 (**c**) and FVB/N (**d**) mice, expressed as time to remove adhesives (s); change in body weight for the C57Bl/6 (**e**) and FVB/N (**f**) strains of mice, expressed as a change in percentage of the pre-stroke baseline weight. Data are presented as mean ± SEM, with *P < 0.05 vs pre-stroke, ^#^P < 0.05, ^##^P < 0.01, ^###^P < 0.001 vs sham, ^$^P < 0.05 vs 15 min, two-way RM ANOVA followed by Tukey’s multiple comparisons test.

The hanging wire test is a measure of grip strength and motor function post-stroke, with mice expected to fall off the wire faster following a stroke. All C57Bl/6 mice subjected to stroke fall off the wire significantly faster than sham-operated mice at 1-day post-stroke, regardless of the length of light exposure (P < 0.001, Fig. 2a). In comparison, in the FVB/N strain, only mice subjected to 18 or 20 min of light exposure have a hanging wire deficit compared to sham at 1-day post-stroke (P < 0.05, Fig. 2b). Nevertheless, both strains of mouse appear to have a similar amount of motor impairment when the length of light exposure is increased to 18 or 20 min (1-day post-stroke: C57Bl/6: 18 min: 97.5 s ± 30.4 s, 20 min: 85.6 s ± 24.7 s; FVB/N: 18 min: 85.8 s ± 10.1 s, 20 min: 108.2 s ± 29.5 s).

The adhesive removal test is a measure of somatosensory function post-stroke, with the length of time taken to remove the adhesives expected to be longer for mice with more somatosensory impairment. In this test, C57Bl/6 mice subjected to stroke took longer to remove the adhesives compared to sham. Increasing the length of light exposure did not influence performance in this test, with all C57BI/6 mice taking a similar amount of time to remove adhesives.

In contrast, in the FVB/N strain, all mice took significantly longer than sham to remove adhesives at 1 day post-stroke, and maintained this deficit at 7-days post-stroke (Fig. 2d). Overall, FVB/N mice appear to have a greater sensorimotor deficit in this test compared to C57Bl/6 mice, with FVB/N mice exposed to 20 min of light taking 33.3 s ± 7.8 s to remove the adhesives at 1-day post-stroke. This is longer than the performance seen in the corresponding C57BI/6 group at the same time (20 min: 16.7 s ± 5.3 s).

In both strains of mouse, regardless of the length of light exposure, mice lost a significant amount of weight across the 7-day testing period compared to sham, with C57Bl/6 mice losing more weight at 1-day post-stroke than FVB/N mice (1 day: C57Bl/6: 15 min 8.97% ± 1.09%, 18 min: 9.42% ± 0.42%, 20 min: 9.10% ± 0.51%; FVB/N: 15 min: 5.49% ± 0.659%, 18 min: 7.06% ± 0.56%, 20 min 6.56% ± 0.62%; Fig. 2e,f. 15-and 18-min: P < 0.005, 20-min: P < 0.05). Interestingly, FVB/N mice subjected to sham surgery lost a significant amount of weight at 1- (3.04% ± 0.79%) and 3-days (4.196% ± 1.04%, P < 0.05) post-surgery (Fig. 2f, P < 0.05), however, this surgery effect was not observed in the C57Bl/6 strain (1-day 1.89% ± 0.752%, 3-days: 0.794% ± 1.121%, Fig. 2e).

### FVB/N mice have heavier spleens than C57Bl/6 mice following stroke

FVB/N mice subjected to stroke have heavier spleens than C57Bl/6 mice (C57Bl/6: 15 min: 2.59 ± 0.07, 18 min: 2.81 ± 0.14, 20 min: 2.65 ± 0.16; FVB/N 15 min: 3.37 ± 0.14, 18 min: 3.77 ± 0.40, 20 min: 3.56 ± 0.14; Supplementary Fig. S2). Additionally, FVB/N mice given 18 or 20 min of light exposure have a significantly larger spleen/body weight ratio compared to sham (FVB/N sham: 2.99 ± 0.1; Supplementary Fig. S2). However, there is no stroke-induced difference in thymus weight between the two strains (Supplementary Fig. S2).

### Cellular architecture

**Figure 3:**
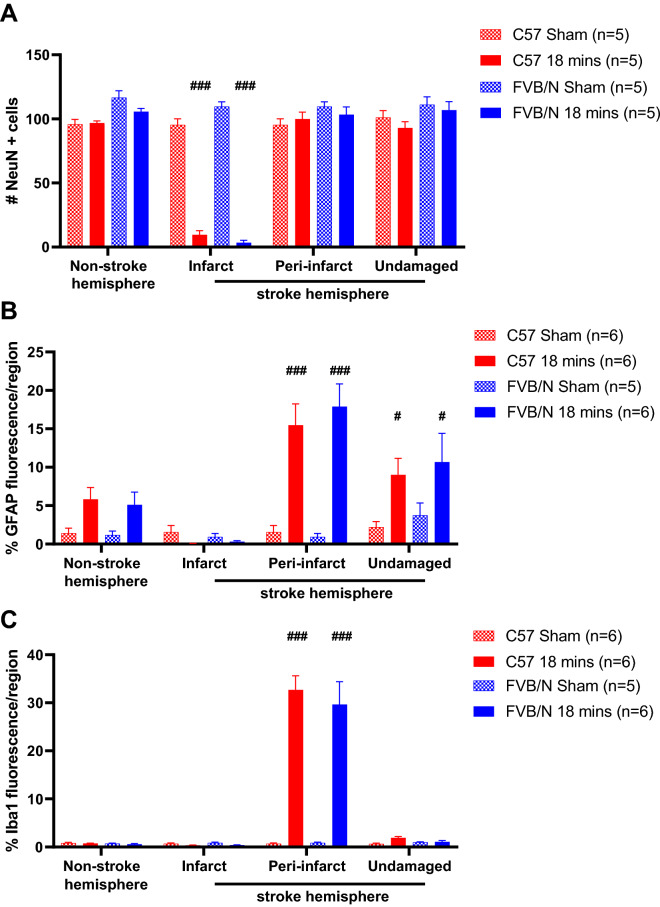
The mean number of NeuN + cells per region, illustrating the number of viable neurons (**A**); the mean percentage of fluorescence per region illustrating the amount of GFAP + staining for reactive astrocytes (**B**); the mean percentage of fluorescence per region of Iba1 + staining for microglia (**C**) in the non-stroke hemisphere, and the infarct, peri-infarct and undamaged regions of the stroke hemisphere in C57Bl/6 and FVB/N mice subjected to 18 min of light exposure during photothrombosis, or sham operated non-stroked mice. Data are represented as mean ± SEM, with ^#^P < 0.05, ^###^P < 0.001 vs sham, two-way ANOVA followed by Tukey’s multiple comparisons test.

In sham-operated mice, there is a consistent number of neurons across the different regions of the brain. However, in mice subjected to stroke, there is a loss of neurons within the brain region directly affected by stroke (infarct region) (Infarct: C57Bl/6: sham: 95 cells ± 5 cells, 18 min: 10 cells ± 3 cells; FVB/N sham: 110 cells ± 4 cells, 18 min: 3 cells ± 2 cells; P < 0.001, Fig. 3A), but not on the peri-infarct region.

PT stroke causes an increase in astrocyte (GFAP) expression within the peri-infarct region of the brain compared to sham (C57Bl/6: sham: 1.56% ± 0.87%, 18 min: 15.47% ± 2.78%; FVB/N sham: 0.94% ± 0.46%, 18 min: 17.896% ± 2.95%, P < 0.001, Fig. 3B). There was also an increase in GFAP staining, albeit to a lesser extent, in the undamaged region of the brain on the stroked hemisphere, (undamaged: C57Bl/6 sham: 2.21% ± 0.72%, 18 min: 9.02% ± 2.14%; FVB/N sham: 3.74% ± 1.6%, 18 min: 10.67% ± 3.74%; P < 0.05, Fig. 3B). Interestingly, there was a trend for an increase in the non-stroked hemisphere compared to sham, although this was not significant (Fig. 3B).

Similarly, microglial (Iba1 +) expression was also increased in the peri-infarct region of the brain, however unlike astrocytes, this staining was specific to this region, with all other regions exhibiting almost no microglia (Peri-infarct: C57Bl/6: sham: 0.68% ± 0.19%, 18 min: 32.68% ± 2.94%; FVB/N sham: 0.85% ± 0.16%, 18 min: 29.64% ± 4.74%; P < 0.001, Fig. 3C). An identical pattern of staining was observed with macrophages (F4/80), with an increase in staining visible in the peri-infarct region, (Infarct hemisphere: C57Bl/6: sham: 0.97% ± 0.50%, 18 min: 14.04% ± 0.99%; FVB/N: sham: 1.56% ± 1.05%, 18 min: 9.41% ± 1.59%; P < 0.001. Supplementary Fig. S6). Furthermore, initially there appeared to be an increase in macrophages (F4/80) staining in the C57Bl/6 strain compared to the FVB/N strain (Infarct hemisphere: C57Bl/6: 14.04% ± 0.99% vs FVB/N: 9.41% ± 1.59%; Supplementary Fig. S6), this difference was lost once the area of staining is normalised to the infarct volume (C57Bl/6: 1.14% ± 0.12% vs FVB/N: 0.94% ± 0.19%; Supplementary Fig. S6).

**Figure 4:**
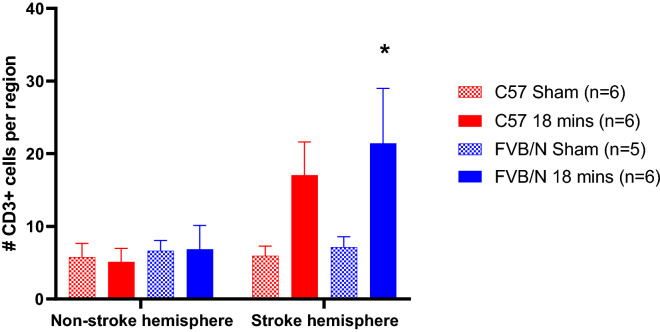
The total number of CD3 + cells per hemisphere of the brain in C57Bl/6 and FVB/N mice subjected to 18 min of light exposure during photothrombosis, or sham operated non-stroked mice. Data are represented as mean ± SEM, with *P < 0.05 vs non-stroke hemisphere, two-way ANOVA followed by Sidak’s multiple comparisons test.

Overall, the expression of CD3 + T cells was low in both sham-operated and stroked mice. There was an increase in T cells in the stroke hemisphere compared to the non-stroked hemisphere, although this is only significant in the FVB/N strain (C57 18 min: non-stroked hemisphere: 5 cells ± 2 cells, stroke hemisphere: 17 cells ± 5 cells; FVB/N 18 min: non-stroke hemisphere: 7 cells ± 3 cells, stroke hemisphere: 21 cells ± 8 cells; Fig. 4).

Overall, there was no difference in any of the cellular markers between the two strains of mice following stroke.

### FVB/N mice have greater BBB breakdown compared to C57Bl/6 mice after 18 min of PT

**Figure 5:**
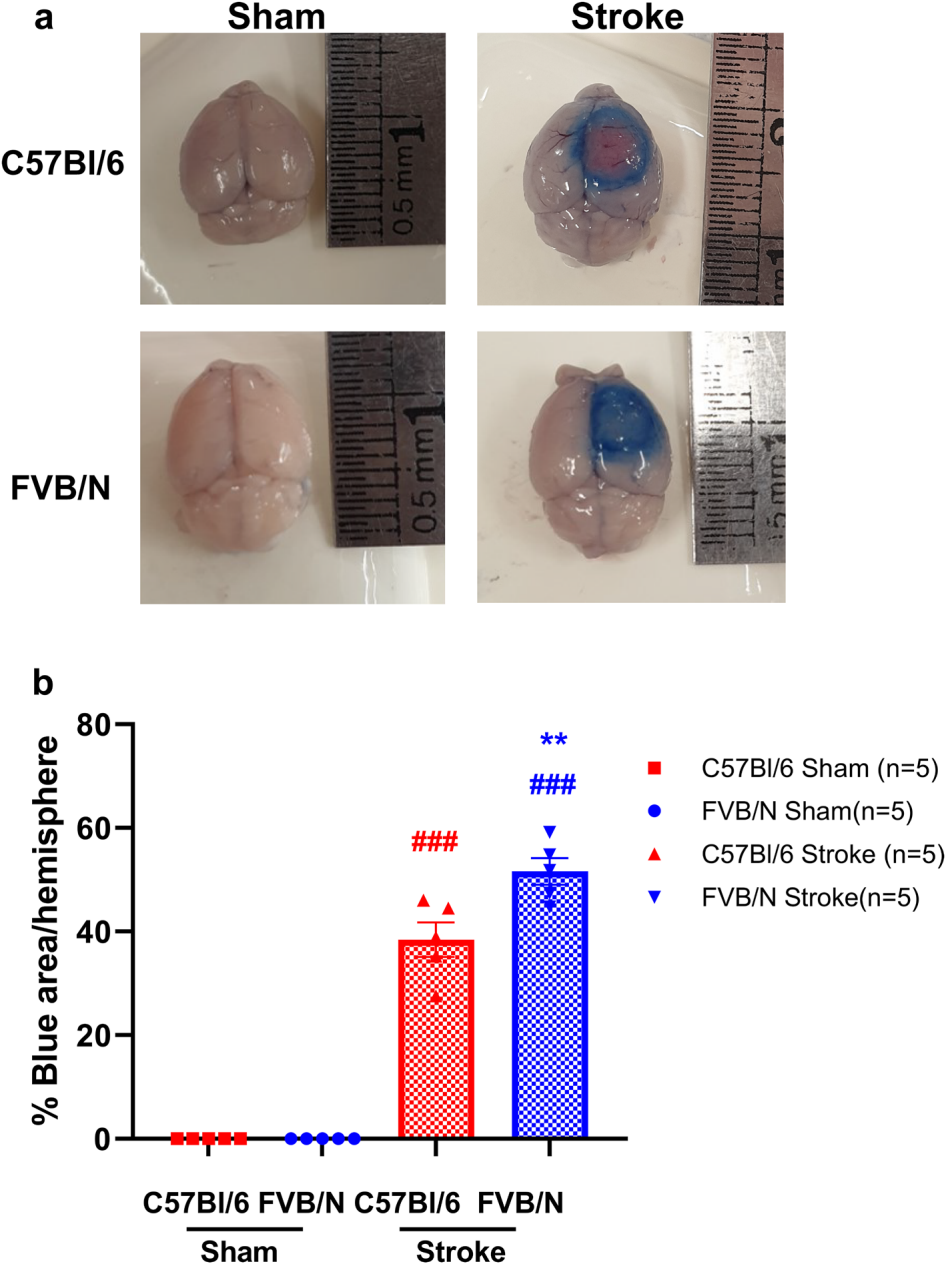
FVB/N mice have greater BBB breakdown compared to C57Bl/6 mice after 18 min of photothrombosis. Representative images (**a**) and quantification (**b**) of evans blue dye extravasation in the brain following 18 min of photothrombosis or sham surgery in the C57Bl/6 and FVB/N strains of mice. ^###^P < 0.001 vs sham, **P < 0.01 vs C57Bl/6, one-way ANOVA followed by Tukey’s multiple comparisons test.

Mice subjected to sham surgery show no evidence of evans blue extravasation into the brain, whereas in the mice subjected to stroke, there is a clear increase in evans blue staining specifically around the region of damage. FVB/N mice have a greater percentage area of evans blue dye in the stroked hemisphere compared to C57Bl/6 mice (C57Bl/6: 38.44% ± 3.34% vs FVB/N: 51.61% ± 2.57%; P < 0.01, Fig. 5). In the C57Bl/6 mice, the evans blue dye leakage was confined to the peri-infarct region of the brain, whereas there was leakage throughout the infarct and peri-infarct regions in brains taken from FVB/N mice (Fig. 5).

## Discussion

The current study demonstrates that increasing the length of light exposure progressively increases the extent of damage in the brain, with longer light exposures resulting in larger infarct volumes (Fig. 1). However, this worsening of brain damage was not correlated with more severe behavioural deficits, such that there was a disparity between infarct volume and functional outcomes. Interestingly, while there was no difference in infarct volume between the two strains of mouse used, there was a strain-related difference in performance on function tests. FVB/N mice performed better on the hanging wire test but showed a larger stroke-induced deficit using the adhesive removal test compared to C57Bl/6 mice (Fig. 2), indicating that the adhesive removal test may be a better indicator of stroke damage in the FVB/N strain of mouse. While the FVB/N mouse strain exhibited a more pronounced BBB breakdown compared to C57Bl/6 mice (Fig. 5), this did not translate into enhanced immune cell infiltration into the brain.

In different models of stroke, the severity of brain damage can be manipulated. For example, in the filament model of MCAO, increasing the length of time the filament is in situ causes a more severe stroke^24^. Similarly, in the endothelin-1 model of MCAO, it is possible to increase the volume of stroke damage by increasing the amount of endothelin-1 administered^24^. The current study has shown that stroke severity can be altered by changing the length of light exposure. The PT model involves the formation of clots in the microvasculature, which are driven by the interaction between light and rose bengal^5^. Extending the length of light exposure increases the reaction time, which presumably amplifies the clot formation within the microvasculature, which in turn would increase the area with reduced cerebral blood flow, and the infarct volume. The ability to manipulate the volume of damage diversifies this model of stroke and presents the possibility that this method of stroke induction could be used to target different brain regions, or to represent a more severe stroke. This may also be achieved by manipulating the power of the light source if possible. Indeed, it has been shown that changing the power of a light source without changing the duration of light exposure can cause differences in infarct size^25^. However, whilst increasing light exposures resulted in greater infarct volumes, this did not translate to a worsening of the physical symptoms of stroke.

Increasing the length of light exposure did not increase functional deficit in C57Bl/6 mice, despite there being an increase in infarct volume (Figs. 1 and 2). This disparity between infarct volume and behavioural performance has been previously reported in this model of stroke in mice, such that mice with a larger region of damaged tissue do not necessary have poorer outcomes physically^9^. The fact that increasing light exposure does not translate into a generalised worsening of the functional outcomes, suggests that even the shortest length of light exposure (15 min) completely damages the primary motor cortex (M1) and somatosensory cortex (S1), which are the regions of the brain involved in determining performance on both tests used in the current study. The increase in infarct volume facilitated by lengthening the time of light exposure most likely causes damage in regions adjacent to M1 and S1 which are not involved in performance on either the hanging wire or adhesive removal test. Additionally, a limitation of the current study is that the functional tests performed do not detect asymmetries in motor performance, which would arise from a unilateral stroke. It is possible that the dominant, non-stroke hemisphere, may therefore compensate for the stroke damage, although that would require that the unimpaired side responds differently across treatment groups which seems unlikely. Indeed, similar tests have been used in numerous studies to detect functional stroke impairments^12,26,27^. Nevertheless, it is possible that a wider range of tests are needed to correctly predict behaviour according to infarct volume or vice versa, such as the cylinder test, which is sensitive to unilateral forelimb use^28^.

It is known that mice have strain specific responses to injury^29^, which may influence stroke outcomes. It is also understood that different mouse strains have different responses to stroke^30^. Indeed, the current study clearly demonstrates that FVB/N mice have better functional outcomes in the mild PT model of stroke compared to C57Bl/6 mice. Specifically, FVB/N mice had preserved motor function according to the hanging wire test following a mild stroke (15-min light exposure) compared to their C57Bl/6 counterparts who had a significant deficit (Fig. 2a,b). This strain specific performance on the hanging wire test has also been demonstrated in the filament MCAO model of stroke. Following a 30-min occlusion of the MCAO, FVB/N mice are reported to have less deficit than C57Bl/6 mice on the hanging wire test, despite both strains having the same infarct volume^12^. In contrast, the FVB/N had much more variable performance on the adhesive removal test and appeared to have an exaggerated stroke induced deficit on this test compared to the C57Bl/6 mice (Fig. 2c,d). An advantage of this study is that all experiments were performed by the same experimenter and under identical laboratory conditions, meaning that any differences in outcomes between these strains are due to a strain-effect, and not differences between surgeons or experimenters when handling animals. This clearly highlights the need to choose tests which are appropriate for the strain of mouse being used, for example, the hanging wire test is more sensitive to the stroke effect in C57Bl/6 mice, if 15-min of light exposure is to be used, whereas the adhesive removal test may be better suited to FVB/N mice.

As expected, C57Bl/6 mice lost more weight post-stroke compared to FVB/N mice (Fig. 2e,f), which has been previously reported in the MCAO model of stroke^12^. Surprisingly, FVB/N mice experienced surgery-induced weight-loss, such that even the sham group in the FVB/N strain lost a significant amount of weight over the experimental period (Fig. 2f). This surgery related weight loss is unlikely to be related to anaesthesia, as it has been previously shown that there is no strain specific susceptibility to isoflurane^31^. It is possible that the surgery-induced weight loss is a result of increased anxiety levels impacting feeding behaviour post-surgery. Indeed, it has been shown that FVB/N mice experience more stress-induced weight loss than C57Bl/6 mice after 2 h of daily restraint over a 10-day period^32^.

Despite the PT stroke being a mild model of stroke, there was a robust loss of neurons, as evident by a reduction in NeuN + staining, in the infarct region. As expected, this loss of neurons was specific to the infarct region only, with all other regions, and sham animals having high neuronal staining (Fig. 3A). Both strains of mice had similar numbers of surviving neurons, which is consistent with having similar infarct volumes. Similarly, there was also a decrease in astrocytes and microglia in the infarcted region, which combined with the lack of neurons, suggests that this area has undergone necrosis (Fig. 3). Furthermore, consistent to what has previously been reported in this model of stroke, astrocytes, macrophages and microglia were all upregulated in the peri-infarct region ^26^, indicating that these cells are all involved in the endogenous response to stroke. There were also no differences in the amount of positive staining for reactive astrocytes, macrophages, or microglia between the two strains (Fig. 3 and Supplementary Fig. S6). Interestingly, there was an increase in positive staining for astrocytes across the whole brain, even in regions remote to the site of injury, suggesting that even in a mild, focal model of stroke, the response to injury is a global event involving the entire brain.

Overall, we observed a low expression of T cells after photothrombosis, with a small increase in the number of T cells in mice subjected to stroke within the affected hemisphere (Fig. 4). Whether such low numbers of cells are likely to have a physiological impact is unclear. We did observe a slight increase in T cell levels in FVB/N mice compared to C57Bl/6 mice subjected to stroke, which is the opposite to what has been previously shown^12^. It is possible that strain specific differences in the cellular responses to stroke seen previously were not observed because the current stroke model is not severe enough to produce a robust immune response. For noting, the previous study examined immune cells at 24-h post-stroke, whereas our study looked at the cellular responses at 7-days post-stroke. It is generally accepted that many inflammatory markers will increase up to 3 days post stroke, and then will gradually diminish^33^, thus in the future it may be beneficial to examine inflammation in a more acute setting, for example at 24 h post-stroke.

There is a greater increase in spleen size in the FVB/N mice following stroke compared to C57Bl/6 mice, despite the fact that both strains have similar spleen weights in the sham groups (Supplementary Fig. S2). In addition, the FVB/N mouse had a greater degree of blood brain barrier (BBB) breakdown than C57Bl/6 mice (Fig. 5). This is the first time a difference in the BBB breakdown between these strains has been observed. Surprisingly, this did not correlate with changes in infarct volume, functional outcomes, or direct measurement of immune cell infiltration. However, BBB breakdown was measured at 4.5-h post-stroke, whereas the other endpoints were assessed at 7-days post-stroke. It is possible that by the 7-day time point, any differences between the two strains had resolved. Indeed, it has been shown that FVB/N mice have elevated levels of neutrophils in the filament model of stroke^12^, which is consistent with the elevated breakdown of the BBB, however, neutrophil levels were measured at 24-h post-stroke. Additionally, the increased spleen size in FVB/N mice post-stroke also suggests a difference in immune response, which may contribute to the increase in BBB breakdown in this strain compared to C57Bl/6 mice.

In conclusion, we have shown that increasing the length of light exposure during PT stroke causes an increase in infarct volume, but this does not translate into a worsening of functional outcome post-stroke. There are strain specific differences in functional outcomes, with the FVB/N strain of mouse being more resilient against the motor symptoms of stroke, but more sensitive to stroke impact on somatosensory tasks. Similarly, there are strain specific differences in BBB breakdown and spleen size, however, this was not associated with a difference in cellular response to stroke. Now we have more extensively characterised the PT model of stroke, it opens the opportunity to vary stroke severity with this model using different lengths of light exposure. Given that there are limited stroke models and that there has been a failure to translate pre-clinical studies into humans, being able to use photothrombotic stroke to model different levels of stroke severity or location may help improve this issue with translation.

## Supplementary Information

Supplementary Information.

## Data Availability

All data generated or analysed during this study are included in this published article and its supplementary information files.
