# Supplementary material for: Optimising the photothrombotic model of stroke in the C57BI/6 and FVB/N strains of mouse
Source: Sci Rep. 2022 May 9;12:7598. doi: 10.1038/s41598-022-11793-6 (PMC9085761; doi:10.1038/s41598-022-11793-6)
Supplement: Supplementary file 1 — Supplementary Information. [file 41598_2022_11793_MOESM1_ESM.pdf]

Supplementary Information

Optimising the photothrombotic model of stroke in the C57Bl/6 and FVB/N strains of mouse

Authors: Adriana Knezic, Brad R.S. Broughton, Robert E. Widdop, Claudia A. McCarthy

Photothrombotic stroke induction

Mice were anaesthetised with isoflurane inhalation in an induction box (5% isoflurane; Issorane, Baxter, Baxter Healthcare Pty. Ltd) and then maintained under anaesthesia using a nose cone (1.5% isoflurane). The head was then shaved, a midline incision made over the skull and the head position was secured using a stereotaxic frame. Rectal temperature was monitored and maintained at 37.0 ± 0.5 °C throughout the surgical procedure using an electric temperature controller (Harvard apparatus, Cambridge, UK). An objective lens (5mm), attached to a cold light source (KL 1600 LED, Schott, Mainz, Germany, luminuous flux: 680lm, colour temperature: 5600K) was positioned directly above the M1 region of the left sensorimotor cortex (coordinates: anterior/posterior: 0mm, medial/lateral: +1.5mm). Once the lens was in position, the mouse received an i.p injection of rose bengal solution (0.2ml; 10mg/ml in distilled water; Sigma, St. Louis, USA). After 5 mins, the light source is turned on at maximum power and the M1 region of the brain is illuminated for either 15, 18, or 20 minutes. Following this, the light was switched off, the animal removed from the stereotactic frame, and the incision site closed using skin glue (Vetbond, 3M Animal Care Products, MN, USA). The mouse is then allowed to regain consciousness under a heat lamp before being returned to its home cage. Sham mice were subjected to the same procedure but did not receive the i.p injection of rose bengal. All animals were monitored until 7 days post-surgery.

Immunofluorescence:

The neuronal nuclear marker, NeuN was used to assess the number of viable neurons. Coronal brain sections (10µm thick) were fixed with acetone (100%) for 15 minutes. Following this, sections were washed in 0.01M phosphate buffered saline (PBS; 3X 10 minutes; Gibco). To prevent non-specific binding, sections were incubated with a Mouse on Mouse (MOM) Ig blocking reagent (Vector laboratories, USA) diluted in 10% goat serum (Invitrogen, USA) for 1 hour in a humidified chamber. Sections were then incubated with a mouse anti-NeuN primary

antibody, (1:500; Merck Millipore, USA) diluted in the MOM kit diluent (Vector laboratories, USA), and left at room temperature overnight in a humidified chamber. The next day, sections were washed in 0.01M PBS (3X 10 minutes), and then incubated in the MOM biotinylated anti-mouse IgG reagent (Vector laboratories, USA) at room temperature for 10 minutes. The sections were then washed in 0.01M PBS (3X 5 minutes) and fluorescein avidin DCS (Vector laboratories, USA) was applied for 5 minutes. The sections were then again washed in 0.01M PBS (3X 10 minutes) and were mounted in VECTASHIELD hard set mounting medium with DAPI (Vector laboratories, USA) and coverslipped. Images were captured at 400X magnification using an Olympus fluorescent microscope and CellSens software (Version 1.15; Olympus, USA), 3 images were taken from the stroke hemisphere in the infarct region, peri-infarct region, and undamaged region, and from the non-stroke hemisphere, in the region corresponding to the infarcted area. The number of NeuN positive(+) cells per region were counted using Image J and averaged per region of the brain for each animal.

Changes in immune cell number in the brain post stroke, specifically; astrocytes (GFAP); macrophages F4/80); T cells (CD3); and microglia (Iba1) were also assessed using immunohistochemistry. Coronal brain sections (10µm thick) were fixed with 4% paraformaldehyde (PFA, Sigma) for 15 minutes. Following this, sections were washed in 0.01M phosphate buffered saline (PBS; 3X 10 minutes; Gibco). To prevent non-specific binding, sections stained with the antibodies GFAP and Iba1 were blocked using 5% bovine serum albumin (BSA, Sigma) in PBS and 0.3% Triton-X (Sigma). For the CD3 or F4/80 antibodies, non-specific binding was blocked using 10% normal goat serum (ThermoFisher scientific, USA) for 30 minutes in a humidified chamber. Sections were then incubated with the relevant primary antibodies; goat anti-GFAP antibody, (SAB2500462, 1:500 dilution; Sigma); goat anti-Iba1 antibody (ab5076, 1:400 dilution, Abcam); rat anti-F4/80 antibody (MCA497G, 1:100 dilution, Bio-Rad); or rabbit anti-CD3 antibody (Ab16669, 1:200 dilution, Abcam) diluted in the DAKO antibody diluent (DAKO, USA) and left at room temperature overnight in a humidified chamber. The next day, sections were washed in 0.01M PBS (3X 10

minutes), and then incubated with the appropriate secondary antibodies: Alexa fluor 488 donkey anti-goat IgG (GFAP and Iba1), 488 goat anti-rat (F4/80), 594 goat anti-rabbit (CD3) (1:500 dilution; Thermo Fisher Scientific) for 2 hours. The sections were then washed in 0.01M PBS (3X 10 minutes) and were mounted in VECTASHIELD hard set mounting medium with DAPI (Vector laboratories, USA) and coverslipped. Images were captured at 400X magnification using an Olympus fluorescent microscope and CellSens software (Version 1.15; Olympus, USA). For GFAP and Iba1, 3 images were taken from the infarct region, peri-infarct region and undamaged region in the stroked hemisphere. In the non-stroke hemisphere, images were taken in the region corresponding to the infarct region on the stroked hemisphere. The percentage area of positive staining of GFAP or Iba1 was measured using Image J and averaged per region of the brain for each animal. For F4/80, the stroke and non-stroke hemispheres were imaged at 40X magnification, and the percentage area of positive staining was measured with ImageJ and averaged per region for each animal. For animals subjected to stroke, the amount of fluorescence on the stroke hemisphere was also normalised to infarct volume (% fluorescence/infarct volume). For CD3 staining, images of all CD3 positive cells were taken across the whole stroked and non-stroked hemispheres. The number of cells in each of these regions were counted and averaged per brain hemisphere for each animal.

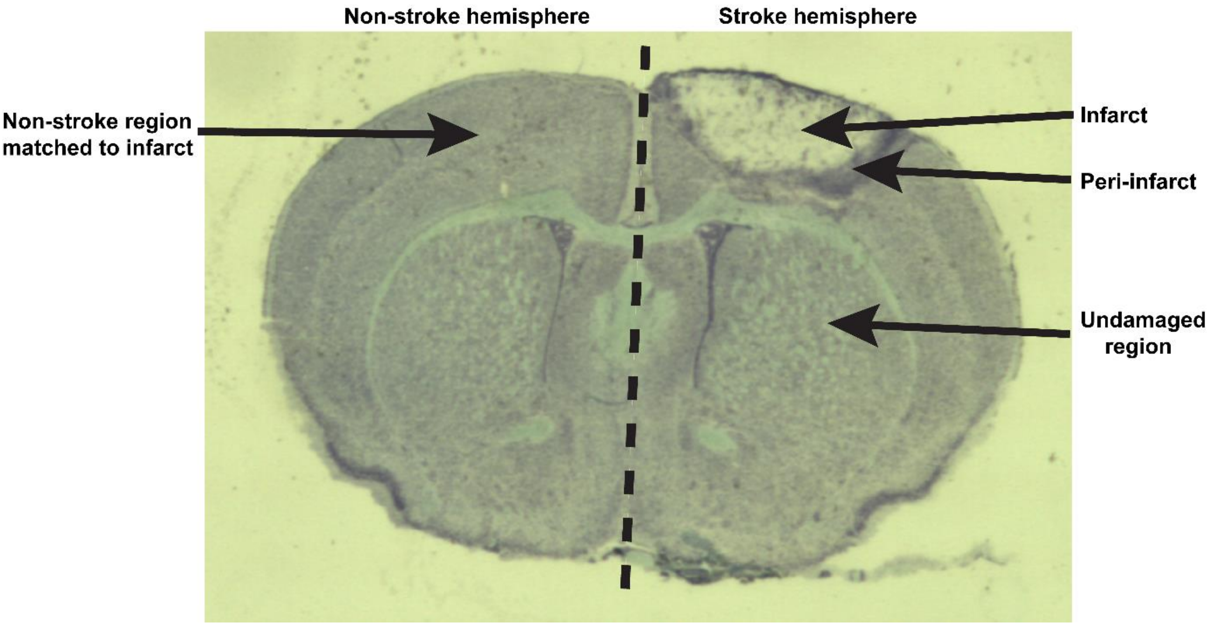

Supplementary Figure S1. Thionin stained brain showing the different regions imaged for immunofluorescence.

Online Figures

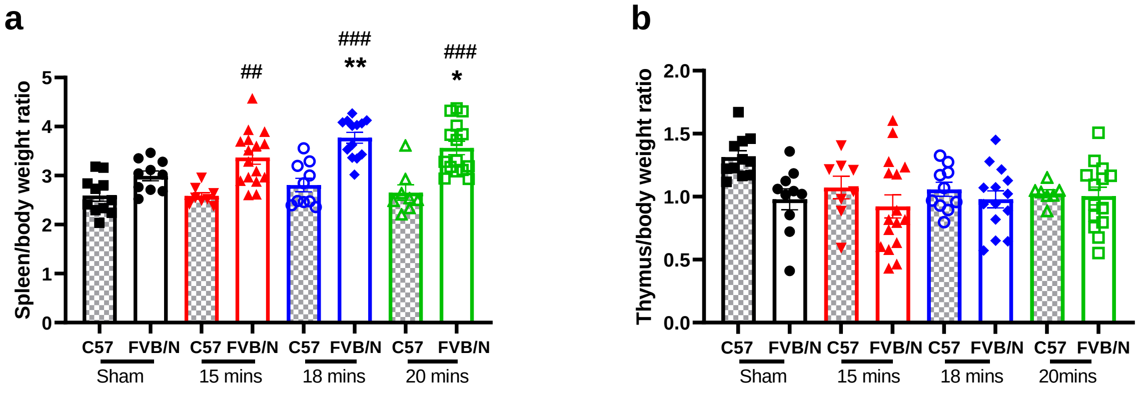

Supplementary Figure S2. Effects of light exposures of 15, 18, and 20-minutes during stroke induction, as well as sham operated non-stroked mice on spleen (a) and thymus (b) size, expressed as a ratio of body weight. Data are presented as mean ± SEM, with *P<0.05, **P<0.01 vs sham, ##P<0.01, ###P<0.001 vs C57, one-way ANOVA followed by Tukey's multiple comparisons test.

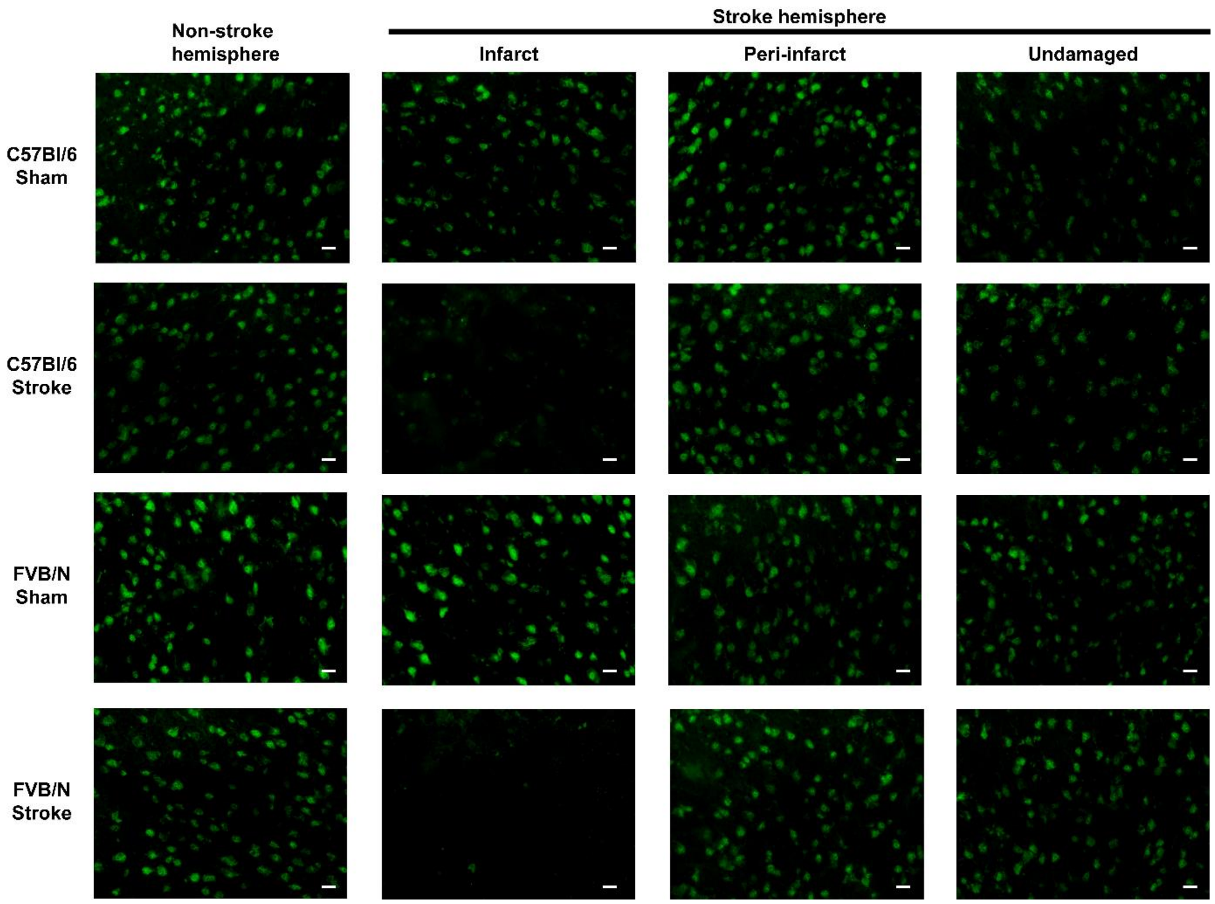

Supplementary Figure S3. Representative images of NeuN immunofluorescence, illustrating neuronal survival in brain regions of C57Bl/6 and FVB/N mice subjected to sham-surgery or 18 minutes of photothrombosis (400X magnification). Scale bar represents 20µm.

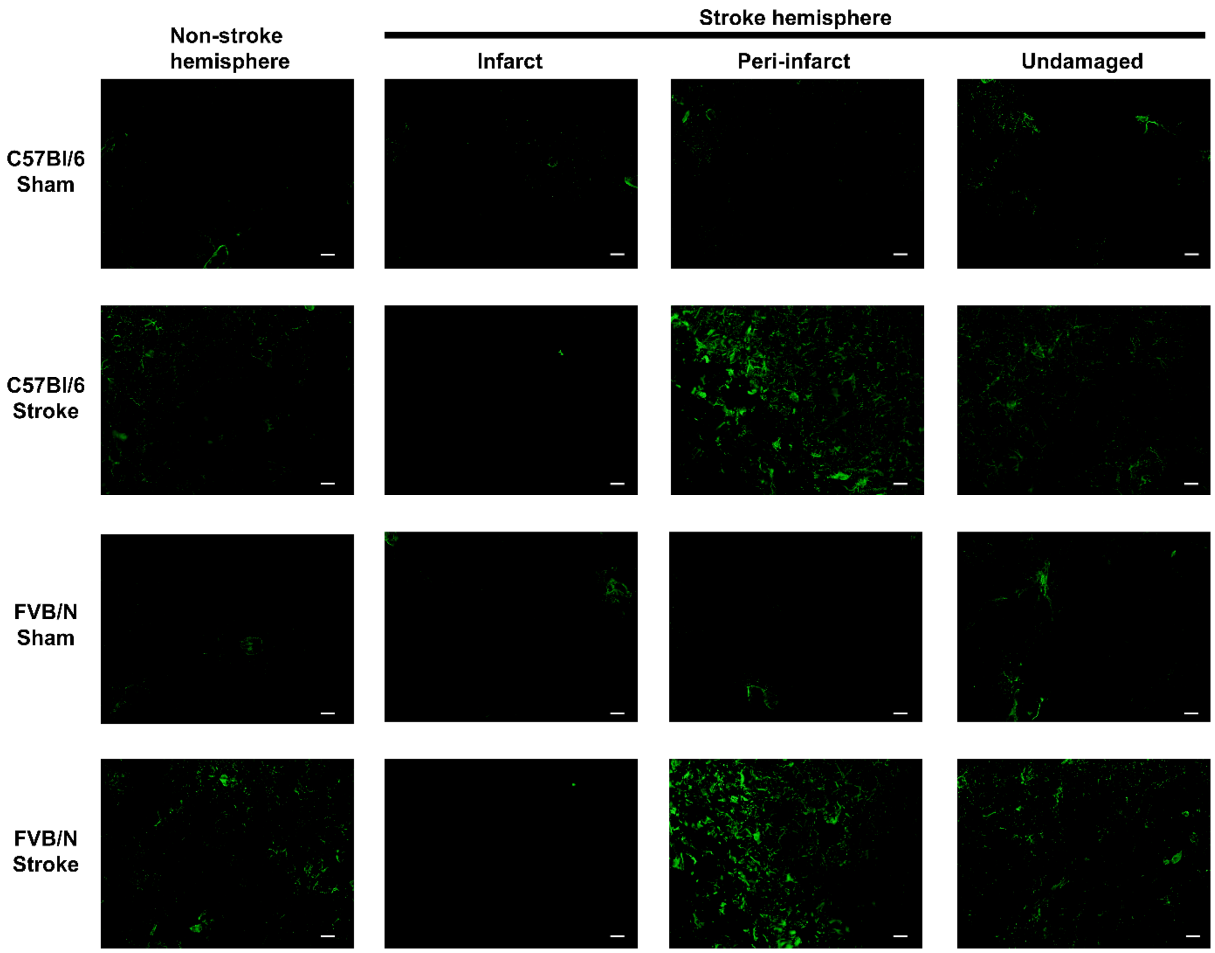

Supplementary Figure S4. Representative images of GFAP immunofluorescence, illustrating astrocytes in various brain regions of C57Bl/6 and FVB/N mice subjected to sham-surgery or 18 minutes of photothrombosis (400X magnification). Scale bar represents 20µm.

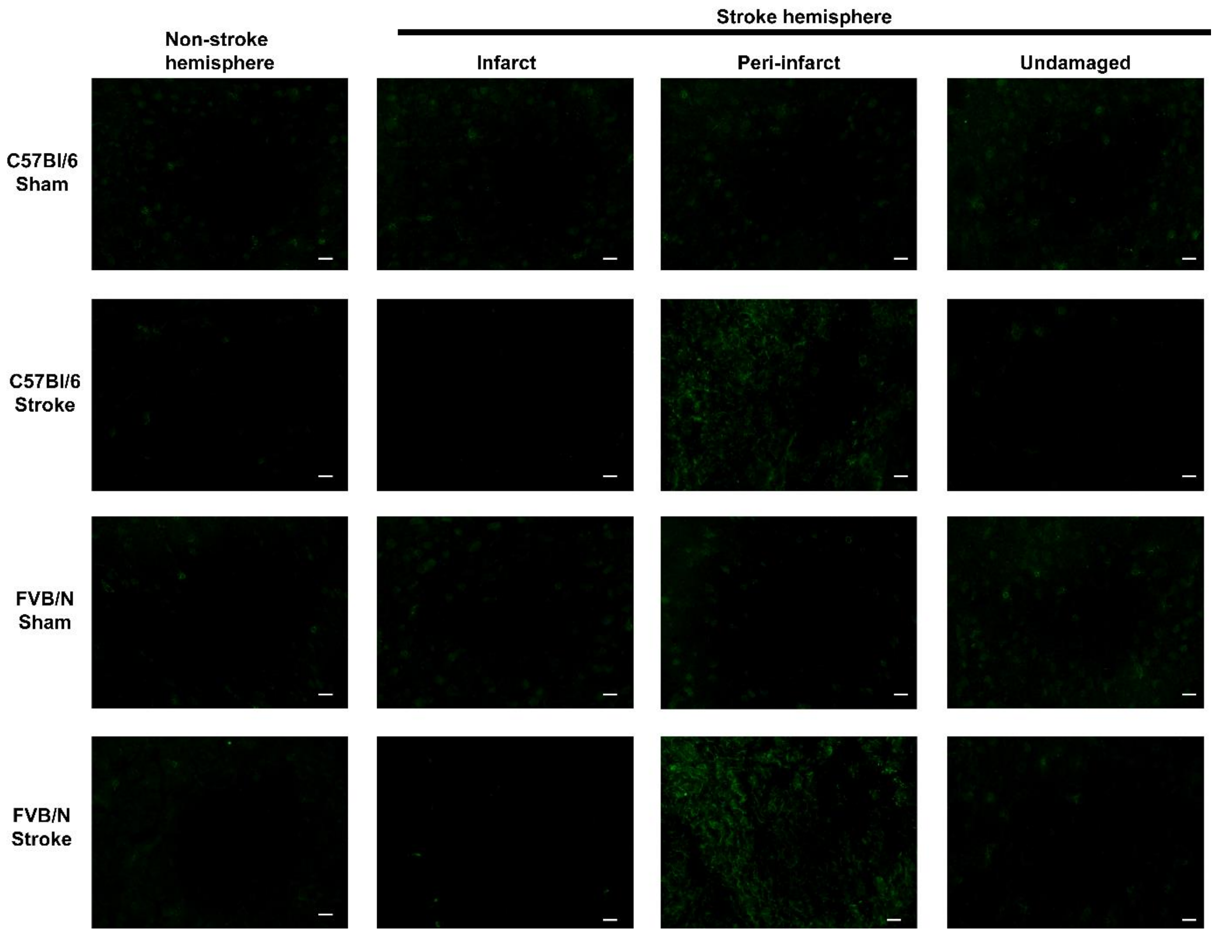

Supplementary Figure S5. Representative images of Iba1 immunofluorescence, indicating microglial activation in various brain regions of C57Bl/6 and FVB/N mice subjected to sham-surgery or 18 minutes of photothrombosis (400X magnification). Scale bar represents 20µm.

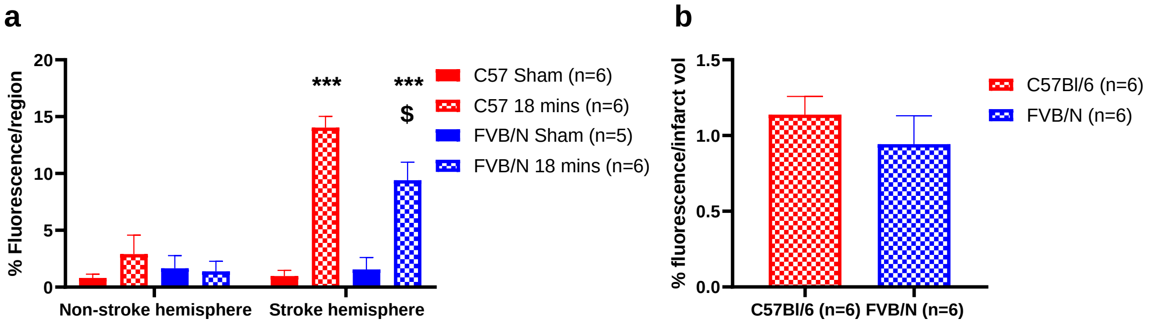

Supplementary Figure S6. Mean data showing (a) the percentage of F4/80 positive staining for macrophages in the infarcted and contralateral hemispheres of the brain and (b) the amount of fluorescence normalised to the infarct volume in C57Bl/6 and FVB/N mice subjected to 18 minutes of light exposure during photothrombosis (FVB/N n=6, C57Bl/6 n=6), or sham operated non-stroked mice (FVB/N n=5, C57Bl/6 n=6). Data are represented as mean ± SEM, with ***P<0.001 vs non-stroke hemisphere, $P<0.05 vs C57 18 mins, two-way ANOVA followed by Sidak's multiple comparisons test (a), unpaired t test (b).
